# Effects of Crude Oil/Dispersant Mixture and Dispersant Components on PPAR**γ** Activity *in Vitro* and *in Vivo*: Identification of Dioctyl Sodium Sulfosuccinate (DOSS; CAS #577-11-7) as a Probable Obesogen

**DOI:** 10.1289/ehp.1409672

**Published:** 2015-07-02

**Authors:** Alexis M. Temkin, Robert R. Bowers, Margaret E. Magaletta, Steven Holshouser, Adriana Maggi, Paolo Ciana, Louis J. Guillette, John A. Bowden, John R. Kucklick, John E. Baatz, Demetri D. Spyropoulos

**Affiliations:** 1 Marine Biomedical Sciences Program, and; 2 Department of Pathology and Laboratory Medicine, Medical University of South Carolina, Charleston, South Carolina, USA; 3 Department of Chemistry, Rollins College, Winter Park, Florida, USA; 4 Department of Pharmaceutical Sciences, Medical University of South Carolina, Charleston, South Carolina, USA; 5 Center of Excellence on Neurodegenerative Diseases, University of Milan, Milan, Italy; 6 Department of Obstetrics and Gynecology, Medical University of South Carolina, Charleston, South Carolina, USA; 7 National Oceanic and Atmospheric Administration, and; 8 National Institute of Standards and Technology, Charleston, South Carolina, USA; 9 Department of Pediatrics and Neonatology, Medical University of South Carolina, Charleston, South Carolina, USA

## Abstract

**Background:**

The obesity pandemic is associated with multiple major health concerns. In addition to diet and lifestyle, there is increasing evidence that environmental exposures to chemicals known as obesogens also may promote obesity.

**Objectives:**

We investigated the massive environmental contamination resulting from the *Deepwater Horizon* (DWH) oil spill, including the use of the oil dispersant COREXIT in remediation efforts, to determine whether obesogens were released into the environment during this incident. We also sought to improve the sensitivity of obesogen detection methods in order to guide post-toxicological chemical assessments.

**Methods:**

Peroxisome proliferator–activated receptor gamma (PPARγ) transactivation assays were used to identify putative obesogens. Solid-phase extraction (SPE) was used to sub-fractionate the water-accommodated fraction generated by mixing COREXIT, cell culture media, and DWH oil (CWAF). Liquid chromatography–mass spectrometry (LC-MS) was used to identify components of fractionated CWAF. PPAR response element (PPRE) activity was measured in PPRE-luciferase transgenic mice. Ligand-binding assays were used to quantitate ligand affinity. Murine 3T3-L1 preadipocytes were used to assess adipogenic induction.

**Results:**

Serum-free conditions greatly enhanced the sensitivity of PPARγ transactivation assays. CWAF and COREXIT had significant dose-dependent PPARγ transactivation activities. From SPE, the 50:50 water:ethanol volume fraction of CWAF contained this activity, and LC-MS indicated that major components of COREXIT contribute to PPARγ transactivation in the CWAF. Molecular modeling predicted several components of COREXIT might be PPARγ ligands. We classified dioctyl sodium sulfosuccinate (DOSS), a major component of COREXIT, as a probable obesogen by PPARγ transactivation assays, PPAR-driven luciferase induction *in vivo,* PPARγ binding assays (affinity comparable to pioglitazone and arachidonic acid), and *in vitro* murine adipocyte differentiation.

**Conclusions:**

We conclude that DOSS is a putative obesogen worthy of further study, including epidemiological and clinical investigations into laxative prescriptions consisting of DOSS.

**Citation:**

Temkin AM, Bowers RR, Magaletta ME, Holshouser S, Maggi A, Ciana P, Guillette LJ, Bowden JA, Kucklick JR, Baatz JE, Spyropoulos DD. 2016. Effects of crude oil/dispersant mixture and dispersant components on PPARγ activity *in vitro* and *in vivo*: identification of dioctyl sodium sulfosuccinate (DOSS; CAS #577-11-7) as a probable obesogen. Environ Health Perspect 124:112–119; http://dx.doi.org/10.1289/ehp.1409672

## Introduction

The *Deepwater Horizon* (DWH) oil spill, which began 20 April 2010, resulted in the release of > 200 million gallons of MC252 (Mississippi Canyon block 252) crude oil into the Gulf of Mexico. Approximately 2 million gallons of dispersant was used to emulsify the oil into the water column, with the aim of aiding in oil biodegradation and preventing the oil from reaching fragile nearshore habitats (Kujawinski et al. 2011). The dispersant applied by aerial spray and at the wellhead oil source was primarily COREXIT 9500 (EC9500, EC9500A, and EC9500B) and secondarily COREXIT 9527 (Nalco Environmental Solutions). Dispersant has been shown to increase the bioavailability of oil components, such as polycyclic aromatic hydrocarbons (PAHs) to fishes (Ramachandran et al. 2004). Although many studies in a variety of animal models have focused on the toxicity of crude oil, dispersed oil, or dispersant alone (Almeda et al. 2013; Hemmer et al. 2011; Rico-Martínez et al. 2013), long-term sub-lethal studies are limited.

Both oil and dispersant are implicated as potential endocrine and metabolic disruptors. Crude oil is linked to reproductive effects in male rats (Adedara et al. 2014), and dispersants have been shown to be estrogenic in an *in vitro* transactivation assay using a human liver hepatoma cell line (Judson et al. 2010). Also, maternal exposure to PAHs (major components of oil) in ambient air during pregnancy was associated with a higher prevalence of obesity at 5 and 7 years of age among participants in a New York City birth cohort (Rundle et al. 2012). Given the massive quantity of oil released and the unprecedented use of dispersant during the DWH oil spill, it is important to understand potential impacts to human health through direct and indirect exposures including those resulting from relief efforts and seafood consumption, respectively.

Obesity is a major health problem that contributes to a variety of diseases, including type II diabetes, hypertension, and cancer (Calle 2007; Ogden et al. 2006). Although traditionally attributed solely to an imbalance in energy intake versus expenditure, recent evidence implicates environmental agents known as “obesogens” as potential contributors to the obesity epidemic, especially in children (Grün and Blumberg 2006). The mechanism of action of obesogens is not completely understood, but any chemical that affects food intake, energy expenditure, lipid metabolism, or adipocyte (fat cell) differentiation could potentially act as an obesogen. The master regulator of adipocyte differentiation, the nuclear receptor PPARγ (peroxisome proliferator–activated receptor gamma) (Janesick and Blumberg 2011), is a prime target on which obesogens act. Obesogens include diethylstilbestrol (Newbold et al. 2005), bisphenol A (Somm et al. 2009), various phthalates and their metabolites (Stahlhut et al. 2007), fungicides and insecticides such as triflumizole (Li et al. 2012), hydrocarbons (Tracey et al. 2013), and the marine anti-fouling agent tributyltin (Kirchner et al. 2010). It is therefore relevant to determine whether novel obesogens exist in oil or dispersants.

We investigated the obesogenic potential of COREXIT 9500–dispersed MC252 crude oil and identified the major COREXIT component, dioctyl sodium sulfosuccinate (DOSS), as a likely obesogen. In addition to it being a major component of the dispersant COREXIT, DOSS is widely used in pharmaceuticals and personal care products [U.S. Department of Health and Human Services (DHHS) 2014; Environmental Working Group (EWG) 2015a].

## Materials and Methods

*Transfection and PPAR*γ *reporter gene assays.* For PPARγ transactivation assays, HEK293T/17 cells (ATCC CRL-11268) were maintained in DMEM/F12 (Dulbecco’s Modified Eagle Medium: Nutrient Mixture F-12; Gibco by Life Technologies) containing 10% fetal bovine serum (ThermoScientific), 2 mM glutamax, 100 μM nonessential amino acids, and antibiotic/antimycotic. Cells were transfected with Lipofectamine 2000 according to the manufacturer’s protocol (Invitrogen by Life Technologies) and plated in 96-well dishes at a density of 20,000 cells per well. Each well of cells was transfected with 16 ng PPARγ-Gal4 [fusion protein of the yeast GAL4 DNA-binding domain (amino acids 1-147) and the mouse PPARγ ligand-binding domain (a.a. 163–475); kindly provided by B. Blumberg, University of California, Irvine], 80 ng UASx4 TK-luc [contains four copies of the GAL4 upstream activating sequence and the herpes virus thymidine kinase promoter (–105/+51) driving firefly luciferase] and 4 ng of pRL vector (encoding Renilla luciferase to control for transfection efficiency). The next day, triplicate wells of cells were treated as described. Cell lysates were harvested after 18 hr of treatment and the Dual-Luciferase Reporter Assay System (Promega) and a Veritas microplate luminometer (Turner Biosystems) were used to measure firefly and Renilla luminescence according to the manufacturers’ recommendations. For assays using human PPARγ, PPARα, and PPARβ/δ plasmids were kindly provided by B. Abbott, U.S. Environmental Protection Agency, and are described by Bility et al. (2004). The GAL4-RXRα plasmid was kindly provided by B. Blumberg (Grün and Blumberg 2006).

*Preparation of oil water-accommodated fractions.* To distinguish PPARγ activity originating from the dispersant COREXIT 9500 (Nalco Environmental Solutions LLC) from MC252 oil (AECOM), a suite of water-accommodated fractions (WAF) was prepared using alternative oils and solvents. All water-accommodated fraction mixtures were prepared by vigorously stirring the components overnight followed by 12 hr of gravity separation and aqueous phase collection. CWAF (COREXIT water-accommodated fraction) is COREXIT 9500, MC252 oil, and DMEM/F12 cell culture media mixed 1:20:200 (ratios by volume). WAF is MC252 oil and DMEM/F12 mixed 1:10. C_M_WAF (COREXIT Mazola corn oil water-accommodated fraction) is COREXIT, Mazola corn oil, and DMEM/F12 mixed 1:20:200. COREXIT only mixtures are COREXIT and DMEM/F12 mixed 1:200. DWAF (dimethyl sulfoxide water-accommodated fraction) is DMSO (dimethyl sulfoxide), MC252 oil, and DMEM/F12 mixed 2:20:200.

*Solid-phase extraction (SPE).* Fractionation of CWAF was performed to determine if compounds from COREXIT or oil were responsible for the observed PPARγ activity. Bond Elut 3-mL silica solid phase columns (Agilent Technologies) and vacuum manifold chambers were employed to fractionate CWAF. Four fractions bearing differential polarities and hydrophobicities were collected in the following order: 50:50 water:ethanol, methanol, dichloromethane, and hexanes. For every 75 μL of CWAF loaded into the column, 100 μL of water was used to pull the sample through the column, and 2 mL of solvent was used to collect each fraction. Before downstream applications solvents were removed by vaporization using a Savant ISS110 SpeedVac Concentrator (Thermo Scientific) and resuspended in 10 μL DMSO/500 μL of fraction.

*Chemical analysis of CWAF sub-fractions.* Composition of the CWAF ethanol:water extractable SPE fraction was determined using a heated (50°C) Kinetex liquid chromatography (LC) C-18 column (100 mm × 2.1 mm, 1.7 μm; Phenomenex) on an Agilent 1100 LC with autosampler. The mobile phases were solvent A: milli-Q water with 1.0 M ammonium acetate (pH 6.5; Fisher Scientific) and 0.1% formic acid:water (volume fraction, 98%, ACS grade; EMD Millipore); and solvent B: 1:1 (vol/vol) isopropanol (IPA):acetonitrile (ACN) (volume fraction, Scientific) with 1.0 M ammonium acetate (pH 6.5) and 0.1% formic acid:IPA/ACN (volume fraction). The components of CWAF (5 μL injection) were separated using a flow rate of 200 μL/min that started at 40% solvent A (for 5 min) and changed to 20% A (0 min to 5 min), to 0% A (5 min to 19 min), back to 40% A (19 min to 20 min), and equilibrated at 40% A for 5 min. Separated components were detected using an AB Sciex API 4000 triple quadrupole mass spectrometer (equipped with a TurboV electrospray ionization source) operating with Analyst software (v. 1.5.2). Full-scan (FS) mass spectrometric (MS) experiments (scanning *m/z* 200–1,200) were performed in both positive and negative polarity mode to identify potential target masses in the ethanol/water fraction. The FS parameters (positive/negative) were scan rate (2 sec), entrance potential (10/–10 V), declustering potential (75/–75 V), curtain gas (20), gas 1 (20), gas 2 (20), ion spray voltage (5,000/–4,500 V), source temperature (500°C) and interface heater on. To further characterize the target masses, product ion scans (MS/MS) were collected [collisionally activated dissociation (4), collision energy (30 eV), cell exit potential (15 V)]. Upon identification of commonalities in the fragmentation of the target masses, precursor ion scans (PIS) of *m/z* 307.3, 309.3, and 311.3 (corresponding to fragments of ethyl linoleate, ethyl oleate, and ethyl stearate, respectively) were employed to highlight analyte classes that exhibited specific in-source fatty acid fragments commonly associated with polysorbate (Tween) materials (Hvattum et al. 2012). Noting the recently reported ingredients of COREXIT, tentative identification of two dominant components of the CWAF ethanol/water extract, polysorbate 80 (Tween 80) and DOSS, was made by comparing the acquired mass spectrometric data (MS, MS/MS, and PIS) to previously published reports (Chen et al. 2002; Hvattum et al. 2012; Mathew et al. 2012; Ramirez et al. 2013; Zhang et al. 2012).

To confirm the presence of Tween 80, the sub-fraction was analyzed in positive FS mode, revealing several unique mass profiles with mass differences (between adjacent ions) of 22 and 44 amu, reported to be [M + NH_4_]^+^ and [M + 2NH_4_]^2+^ ions of polysorbate species (Zhang et al. 2012). Product ion scans showed that the *m/z* 309.3 ion was the most frequent fragment for the target masses. Previous reports attributed the *m/z* 309 fragment ion to an in-source loss of specific fatty acid esters (ethyl oleate, for *m/z* 309.3) characteristic of polysorbate species (Hvattum et al. 2012), or more recently to a strong presence of oleate-related species in polysorbate 80 (Tween 80) (Zhang et al. 2012). PIS of *m/z* 309.3 demonstrated several mass spectral profiles relating to sorbitan monooleates (specifically 16–27 polyoxyethylene units, as shown in Supplemental Material, Figure S2A), isosorbide monooleates, and sorbitan dioleates, indicating the presence of Tween 80. The other highly abundant component of the CWAF ethanol/water sub-fraction was DOSS. Initial investigation of the extract in negative FS mode revealed an intense ion at *m/z* 421. A total ion chromatogram performed in negative FS mode is shown in Supplemental Material, Figure S2B, with the peak at 2.45 min largely representing the [M-H]^–^ ion of DOSS. To confirm the presence of DOSS, a product ion scan of *m/z* 421.1 was performed, and the resultant fragmentation profile is shown in Supplemental Material, Figure S2B. Based on previous reports, which indicate the presence of fragment ions *m/z* 81 and *m/z* 227 (Mathew et al. 2012; Ramirez et al. 2013), it was confirmed that DOSS was abundantly present in the 50:50 ethanol:water sub-fraction.

*Molecular modeling of COREXIT components binding to the PPAR*γ *ligand-binding domain.* Molecular modeling was assessed using MOE software (Molecular Operating Environment; Chemical Computing Group, Inc.). Two PDB (Protein Data Bank; http://www.wwpdb.org/) crystal structures, 4EMA and 2HFP, constituting the human PPARγ ligand-binding domain bound to different ligands, were compared. Using MOE’s superposition function, no significant differences were found in the active site of PPARγ [RMSD (root-mean-square deviation) < 2Å]. Because it contained our positive control Rosiglitazone (Rosi), 4EMA was chosen as the model. Ten compounds, including some metabolites, were docked to PPARγ. Molecular parameters were set for maximum energy minimization and Amber12 liquid state. Five compounds that constitute COREXIT, a negative control (17β-estradiol), and a positive control (Rosi) were assessed and virtually synthesized based on liquid state parameters at pH 7.4. The run was set to 30 different poses, with an area for 30 refinements if necessary. After parameters were set, a continuous run of all compounds were docked, and descriptors of binding data were given in ascending order related to E score. The E score gives binding efficiency in terms of energy state, with lower E scores indicating higher affinity. Compounds that had the capability to be cleaved by esterases were assessed with and without the fatty acid chains.

In vivo *bioluminescence imaging.* The “repTOP PPRE-Luc” mouse model [PPAR response element (PPRE)–luciferase mouse], containing a PPAR response element–luciferase reporter transgene (El-Jamal et al. 2013) was used for *in vivo* bioluminescence imaging. C57BL/6J mice (originally obtained from Charles River Laboratories) were bred in the animal facilities at the Medical University of South Carolina (MUSC). All mice were treated humanely and with regard to alleviation of suffering, according to the *Guide for the Care and Use of Laboratory Animals* (National Research Council 2011). Mice were housed up to five per cage in ventilated air racks with *ad libitum* access to water and irradiated rodent chow (Harlan 2018). Temperature was maintained between 21°C and 23°C, humidity maintained 40%, and a 12-hr light:dark cycle. Five groups of three male mice (littermates age 5–6 weeks) were injected i.p. (intraperitoneally) at 0900 hours in the animal housing facility with saline, 10 mg/kg Rosi (Rosiglitazone; Cayman Chemical), or 50 mg/kg DOSS (Dioctyl sodium sulfosuccinate; Sigma Aldrich). Five hours later mice were anesthetized with 2.5% isofluorane (Sigma Aldrich) in air and injected i.p. with 150 mg/kg luciferin potassium salt (Goldbio). An IVIS 200 bioluminescence imaging system and Living image 4.3.1 software were used to quantify bioluminescence according to the manufacturer’s instructions (Caliper Life Sciences). Specifically, mice were placed in the instrument and received 2% isofluorane in air through nosecones to maintain sedation during image acquisition. PPRE-driven bioluminescence of live mice was quantified using images acquired 10 min after luciferin injection with 1-min exposures. While mice were still sedated, they were sacrificed by cervical dislocation, livers were dissected and washed in PBS and then homogenized in lysis buffer, and luciferase activity was determined in liver homogenates using the Luciferase Reporter Assay System and a Veritas microplate luminometer. All animal procedures were approved by the Institutional Animal Care & Use Committee, MUSC (Animal Welfare Assurance #A3428-01).

*TR-FRET PPAR*γ *competitive binding assay.* Competitive time-resolved fluorescence resonance energy transfer (TR-FRET) (Toth et al. 2012) binding assays were used to determine the affinity of DOSS for PPARγ (SelectScreen Service; Life Technologies). For these assays the donor fluorophore (terbium) on the receptor causes energy transfer to the acceptor fluorophore (Fluormone Green) on the bound ligand, resulting in emission at 520 nM. With increasing doses of ligand (e.g., DOSS), there is increasing displacement of receptor-bound Fluormone Green–tagged positive control ligand and, hence, less signal. Known concentrations of test and control ligands allow fluorescent emission loss to be used to quantitate binding affinity. Terbium is excited with a 340-nm filter and emits multiple peaks, the first of which (485–505 nm) overlaps with the maximum excitation peak of Fluormone Green. To measure energy transfer to Fluormone Green without interference from terbium, a 520/25-nm filter is used with a 100-μsec delay and 200-μsec integration.

*Adipogenic differentiation assays.* For triacylglycerol staining assays to quantify adipogenic differentiation, 3T3-L1 preadipocyte cells (Zenbio) were plated in 48-well plates at a density of 10,000 cells per well in preadipocyte growth medium (PGM; DMEM/F12 supplemented as described above) and grown until confluence. Two days postconfluence, the media were changed to minimal induction media [MIM; PGM supplemented with 62.5 nM dexamethasone, 0.125 mM IBMX (3-isobutyl-1-methylxanthine), and 250 ng/mL insulin] and either different concentrations of DOSS (10, 20, 25, or 50 ppm) or 1 μM Rosi. After 72 hr of induction, the media was switched to PGM containing 1 μg/mL insulin. Cells were allowed to differentiate for 3 more days (6 days total) and fixed with 4% paraformaldehyde. Triacylglycerol staining with AdipoRed (Lonza) and nuclear counter-staining with NucBlue (Hoechst; Invitrogen) was conducting according to the manufacturers’ recommendations. Adipogenesis was quantified by mean relative fluorescent units of 42 fields per well (10× magnification) with five replicates used for each treatment using a HERMES high content screening scanner (WiScan; IDEA Bio-Medical Ltd.). Hoechst fluorescence was determined using excitation 390/18 nm and emission 440/40 nm (light intensity: 50%; exposure: 30 msec; gain: 30%), and AdipoRed was quantified using excitation 485/20 nm and emission 525/30 nm (light intensity: 90%; exposure: 58 msec; gain: 30%).

*mRNA expression via quantitative polymerase chain reaction (qPCR).* 3T3-L1 cells were treated with test ligands as described above. After 72 hr of exposure to MIM supplemented with varying concentrations of DOSS or Rosi, three wells of treatment were pooled from each of two experiments for mRNA expression analyses. RNA was isolated using the RNeasy Kit (Qiagen), following the manufacturer’s instructions. Gene expression was assessed in triplicate using 25 ng of RNA per qPCR reaction and the iTaq Universal SYBR Green One-Step Kit following the manufacturer’s instructions (BioRad). Results were normalized to the housekeeping gene *Hprt*. Data are expressed as a fold change compared with the MIM-only control. Primer sequences for the queried genes can be found in Supplemental Material, Table S1.

*Statistical analysis.* All data analyses were performed using GraphPad Prism software (GraphPad Software Inc.). After confirmation of normality and equal variance (using Brown–Forsythe and Bartlett tests), one-way analysis of variance (ANOVA) followed by Dunnett’s multiple comparisons post hoc tests were performed. *p*-Values < 0.05 were deemed significantly different.

## Results

**Figure 1:**
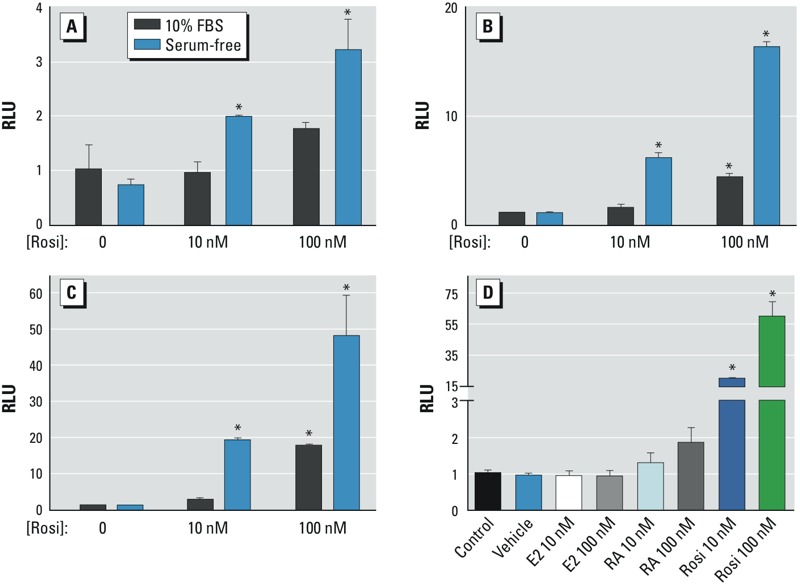
PPARγ transactivation activity in a GAL4-UAS system using serum-free conditions. HEK293T/17 cells were transfected and exposed to the PPARγ agonist Rosi under serum-containing (10% FBS) and serum-free (SF) conditions in triplicate, and luciferase activities were measured following exposure for (*A*) 4 hr, (*B*) 8 hr, and (*C*) 18 hr. Data in *A*–*C* are normalized to the 10% FBS control within each time point. Assay sensitivity is greatly enhanced under SF conditions. (*D*) HEK293T/17 cells were transfected and exposed to estrogen (E_2_), all-trans-retinoic acid (RA), or Rosi under SF conditions, and luciferase activities were measured, demonstrating ligand-specific responsiveness of the system. Data in *D* are normalized to the untreated control. RLU, relative light units. Data are expressed as mean ± SD; *n* = 3 per group (*p < 0.05 vs. control).

*PPAR*γ *ligand-binding assay optimization.* We optimized a rapid, sensitive, specific, and robust system for detecting PPARγ transactivation activity in MC252 oil and COREXIT dispersant. Considering that COREXIT is a mixture of solvents and surfactants that might compromise membrane integrity and also that components of fetal bovine serum (FBS) may have PPARγ agonist activity that would obscure testing, we determined whether a PPARγ ligand-binding domain–GAL4–UAS luciferase system (Forman et al. 1995) could be conducted with cells under serum-free (SF) conditions. HEK293T/17 cells were transfected with plasmids encoding a yeast GAL4 DNA-binding domain–mouse PPARγ ligand-binding domain fusion protein, a yeast UAS (GAL4 upstream activator sequence) driving firefly luciferase, and a constitutively active Renilla luciferase transfection efficiency control. The PPARγ agonist Rosi confirmed the accuracy of the system for detecting PPARγ transactivation activity. As shown in Figure 1, treatment of transfected cells with 0, 10 nM, or 100 nM Rosi for 4 hr, 8 hr, or 18 hr resulted in time- and dose-dependent increases in luciferase activity in both serum-containing (10% FBS) and SF conditions. Notably, the response to Rosi under SF culture conditions was more pronounced than serum-containing cultures at all time points (Figure 1A–C). Conversely, treatment for 18 hr under SF conditions with 17β-estradiol (E_2_) or all-trans-retinoic acid (RA) did not increase luciferase activity, whereas Rosi treatment induced marked luciferase activity (Figure 1D). These data demonstrate the sensitivity and specificity of the PPARγ ligand-binding domain–GAL4–UAS luciferase system. SF conditions and 18-hr treatments were chosen for subsequent experiments aimed at identifying components of oil and COREXIT bearing PPARγ transactivation activity.

**Figure 2:**
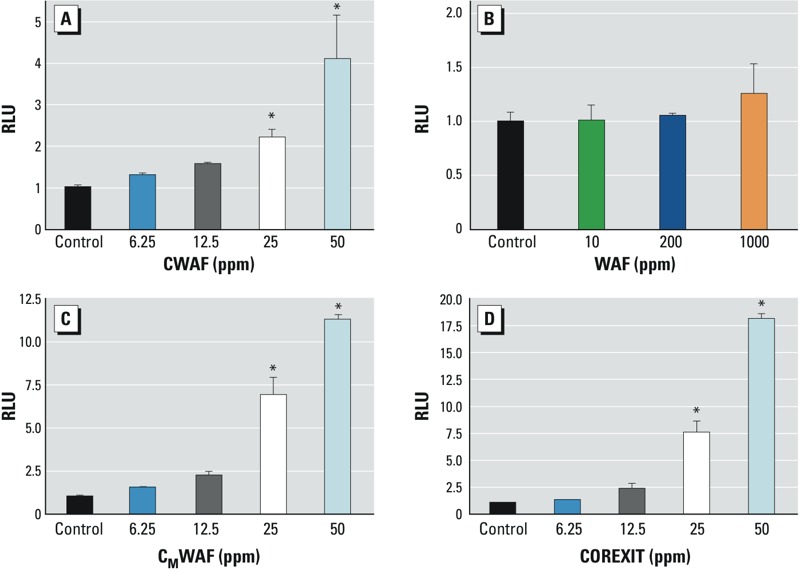
PPARγ transactivation by COREXIT. Dilutions of mixtures were prepared, HEK293T/17 cells transfected and exposed in triplicate for 18 hr, and luciferase activities were measured. (*A*) CWAF, (*B*) WAF, (*C*) C_M_WAF. *A–C* are presented as final concentrations volume fractions. (*D*) COREXIT, diluted in cell culture media to the concentrations indicated volume fractions. Dose-dependent ligand binding activities were detected in CWAF, C_M_WAF, and COREXIT dilutions but not in WAF dilutions. RLU, relative light units.Data are expressed as mean ± SD; *n* = 3 per group (*p < 0.05 vs. control).

*PPAR*γ *transactivation.* To distinguish between PPARγ activity originating from dispersant and from MC252 oil, several mixtures of MC252 oil, with and without COREXIT, were prepared and analyzed for PPARγ transactivation activity, including CWAF, WAF, C_M_WAF, and COREXIT only (see “Materials and Methods”). Dose-dependent PPARγ activation was detected in CWAF, C_M_WAF, and COREXIT dilutions but not in WAF (Figure 2). The CWAF and C_M_WAF fractions comprise the aqueous fraction of the original mixtures of oil and dispersant, and a portion of the amphipathic compounds present in the original mixtures was expected to partition to the organic phase. Also, the dose-dependent PPARγ activation by COREXIT alone (no organic phase) substantially outstripped those of CWAF and C_M_WAF; these results suggest that components of COREXIT were responsible for the activity detected. To further investigate whether oil might have contributed to PPARγ activation by the fractions, an alternate solvent, DMSO, was used to prepare DWAF (DMSO water-accommodated fraction), which lacks COREXIT. Although twice the amount of DMSO was used to prepare DWAF than COREXIT in CWAF and in C_M_WAF, no PPARγ activation was observed in any DWAF dilutions tested (see Supplemental Material, Figure S1), further implicating a COREXIT ingredient and not a component of oil as a PPARγ activator.

**Figure 3:**
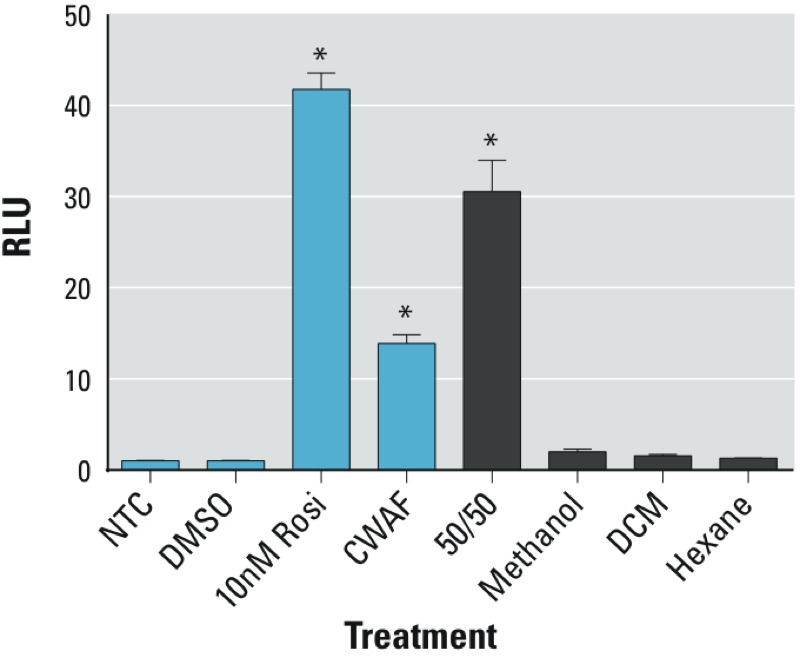
PPARγ transactivation by the 50:50 ethanol:water CWAF volume fraction. CWAF mixtures were fractionated using SPE methods and four solvent types to identify fractions containing PPARγ ligand-binding activity. PPARγ activity was observed only in the 50:50 ethanol:water volume fraction but not for methanol, DCM, or hexane fractions. RLU, relative light units. Data are expressed as mean ± SD; *n* = 3 per group (*p < 0.05 vs. no treatment control).

*CWAF fractionation and analysis.* SPE was employed to separate CWAF into fractions based on polarity and hydrophobicity; CWAF was fractionated into 50:50 water:ethanol, methanol, DCM, and hexane soluble fractions. The PPARγ transactivation system demonstrated substantial PPARγ transactivation activity in the 50:50 water:ethanol fraction, whereas no activity was detected in the methanol, DCM (dichloromethane), and hexane fractions (Figure 3). The compounds present in the CWAF water:ethanol fraction were identified using LC-MS and LC-MS/MS as described in “Materials and Methods.” Analysis of the fraction in positive FS mode resulted in > 200 unique target masses. Tween 80 (polysorbate 80) was concluded to be a highly abundant component of this fraction based on manual inspection and product ion scan profiles (see Supplemental Material, Figure S2A). After investigation of the extract in negative FS mode, DOSS was determined to be another highly abundant component of the CWAF water:ethanol fraction (see Supplemental Material, Figure S2B). Mass spectrometry analyses of the transactivation positive fraction argued that relatively hydrophilic components of COREXIT and not MC252 oil were responsible for the observed PPARγ transactivation activity.

*Modeling of COREXIT components binding to PPAR*γ. Molecular modeling was employed to predict which COREXIT components might bind to PPARγ. Span 80, Tween 80, and DOSS were predicted to bind to the PPARγ ligand-binding domain as shown by their low E scores, whereas propylene glycol and 2-butoxyethanol were not (see Supplemental Material, Figure S3A). Span 80 and Tween 80 have an ester bond that could be cleaved by cellular esterases; once cleaved, neither cleavage product is predicted to bind tightly (see Supplemental Material, Figure S3A). For DOSS docking, the basic lysine 367 residue has a strong hydrogen bond with a sulfonyl oxygen (see Supplemental Material, Figure S3B). The α-carbon adjacent to the sulfonyl group of DOSS shows potential for strong donation to a hydrogen of methionine 364. PPARγ has multiple basic residues that allow pairing with the acidic sulfhydryl group of DOSS, potentially allowing hydrogen bonding between the ligand and receptor. Tween 80, along with other compounds that exhibit low E scores, either have too large a fatty acid group to effectively fit into the binding site of PPARγ or have a charge that is too basic, resulting in higher binding scores (see Supplemental Material, Figure S3A).

**Figure 4:**
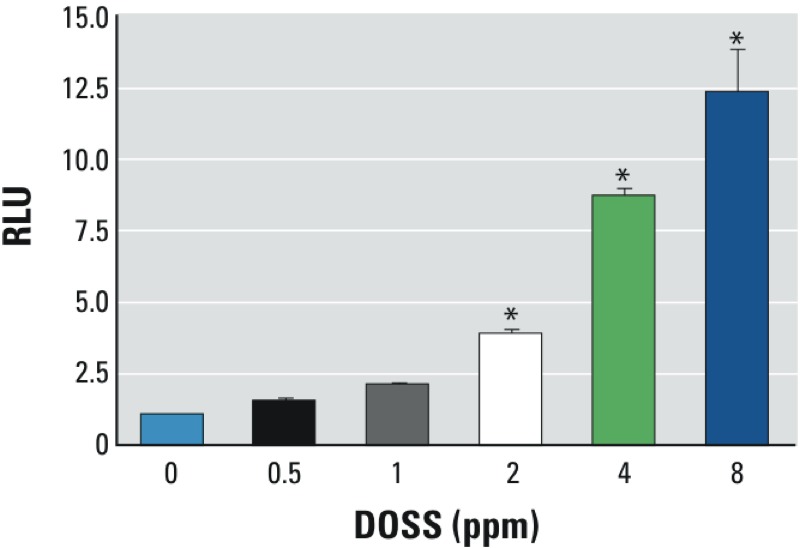
PPARγ transactivation by DOSS. Dilutions of mixtures were prepared, HEK293T/17 cells were transfected and exposed in triplicate for 18 hr, and luciferase activities were measured. Final DOSS concentrations (volume fractions) are indicated. RLU, relative light units. Data are expressed as mean ± SD; *n* = 3 per group (*p < 0.05 vs. vehicle control).

*PPAR*γ *transactivation activity of COREXIT components.* Collectively, the results above implicate a COREXIT component(s) in the PPARγ transactivation observed in the CWAF prepared from MC252 oil. Mass spectrometry indicates that Tween 80 and DOSS are present in the 50:50 water:ethanol fraction of CWAF, which exhibits activity in the transactivation assay, and molecular modeling predicts that Span 80, Tween 80, and DOSS can bind to the PPARγ ligand-binding domain. Because molecular modeling is speculative, these compounds were tested for PPARγ activity. Span 80 did not demonstrate PPARγ transactivation activity even at concentrations much higher than effective COREXIT alone dilutions (see Supplemental Material, Figure S4A). Tween 80 had weak activity that was much too low to account for the activity observed in CWAF and COREXIT (see Supplemental Material, Figure S4B). Furthermore, a mixture of petroleum distillate [ICP (inductively coupled plasma) solvent; CAS (Chemical Abstracts Service) 64742-47-8] and propylene glycol (PG), other major components of COREXIT, did not demonstrate PPARγ transactivation activity (see Supplemental Material, Figure S4C). In contrast, a simplified version of COREXIT containing only ICP, PG, and DOSS demonstrated robust PPARγ transactivation (see Supplemental Material, Figure S4D). DOSS alone elicited dose-dependent increases in PPARγ-driven luciferase expression in the low ppm range (Figure 4). Thus, DOSS has PPARγ transactivation activity, whereas Span 80, ICP, and PG do not. Because COREXIT 9500 is approximately 10% DOSS (Kujawinski et al. 2011), it is likely that the PPARγ agonist activity observed following treatment with CWAF, C_M_WAF, or COREXIT alone is due to DOSS.

**Figure 5:**
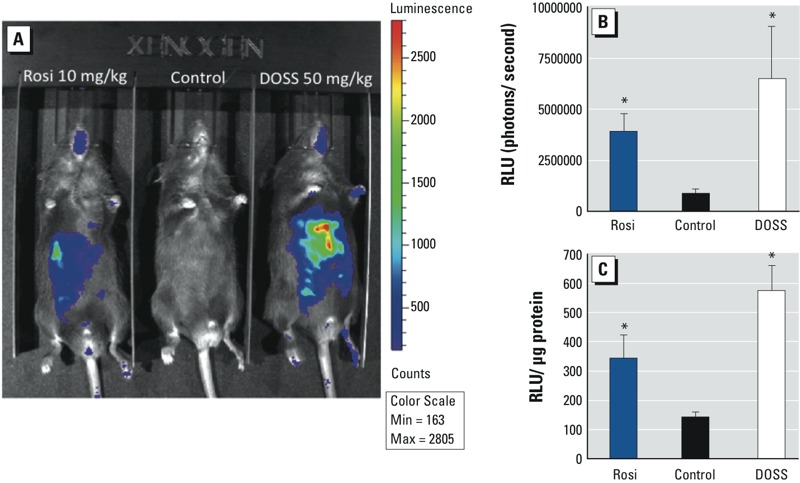
PPRE activity in response to DOSS in PPRE-luciferase mice. (*A*) Representative images of mice treated with 10 mg/kg Rosi, vehicle control, or 50 mg/kg DOSS. (*B*) Luciferase activity of live mice as in *A*. (*C*) Luciferase assay of liver tissue from mice as in *A*. RLU, relative light units. Data are expressed as mean ± SD; *n* = 5 per group (*p < 0.05 vs. vehicle control).

*DOSS PPAR*γ *agonist activity in PPRE-luciferase transgenic mice.* To validate the PPARγ agonist activity of DOSS, PPRE luciferase reporter mice were used as an *in vivo* model. Male littermates age 5–6 weeks were injected with Rosi (positive control), saline (negative control), or DOSS. Imaging from live mice 5 hr post-treatment revealed marked increases in bioluminesence for both Rosi and DOSS treatments and suggested that liver tissue was the main source of the differential luciferase expression, with some expression in the skin of Rosi- and DOSS-treated mice (Figure 5A). To confirm differential PPRE activity in Rosi- and DOSS-treated hepatocytes, liver was dissected and homogenized immediately following imaging and humane sacrifice. As shown, treatment of PPRE-luciferase mice with 10 mg/kg Rosi increased liver luciferase activity approximately 2-fold, whereas treatment with 50 mg/kg DOSS elicited about a 4-fold increase (Figure 5C), demonstrating that DOSS is capable of activating PPAR-driven gene expression in mice. Although PPARα, a major metabolic regulator, is the predominant PPAR isoform in the liver, PPARγ is also expressed in hepatocytes. PPRE activation in the liver opened the possibility that DOSS may activate other PPAR isoforms or RXRα (retinoid X receptor alpha). Transactivation assays using the human LBDs (ligand-binding domain) indicated that 4 ppm DOSS activated PPARγ about 3 fold, whereas activation of PPARα was only about 1.2 fold and nondetectable for the human RXRα LBD (see Supplemental Material, Figure S5A,B,D). Of note, DOSS activated PPARβ/δ about 8 fold at 4 ppm, which is under further investigation (see Supplemental Material, Figure S5C).

**Figure 6:**
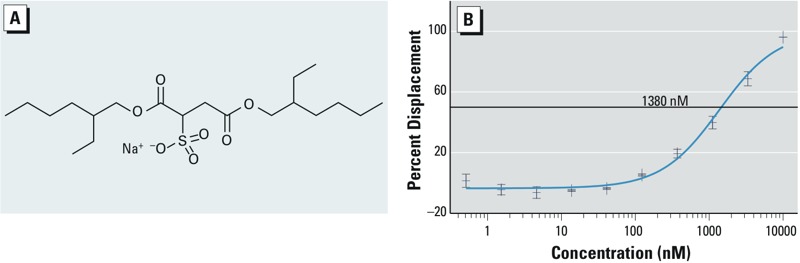
TR-FRET analysis of DOSS binding to PPARγ. (*A*) Molecular structure of DOSS. (*B*) Competitive TR-FRET analysis of DOSS binding to the human PPARγ LBD (apparent K_D_ = 1,380 nM).

*TR-FRET assays of DOSS affinity for PPAR*γ. The structure of DOSS is shown in Figure 6A. The predicted PPARγ receptor-binding affinities are shown in Supplemental Material, Figure S3B. TR-FRET assays were used to measure the affinity of DOSS for PPARγ. As shown in Figure 6B, DOSS binds to the human PPARγ ligand-binding domain with a K_D_ of 1,380 nM. This is a binding affinity comparable to the pharmaceutical PPARγ agonist pioglitazone (K_D_ = 1,310 nM) and the endogenous ligand arachidonic acid (K_D_ = 1,340 nM), as determined in the same assay (Singh et al. 2008).

**Figure 7:**
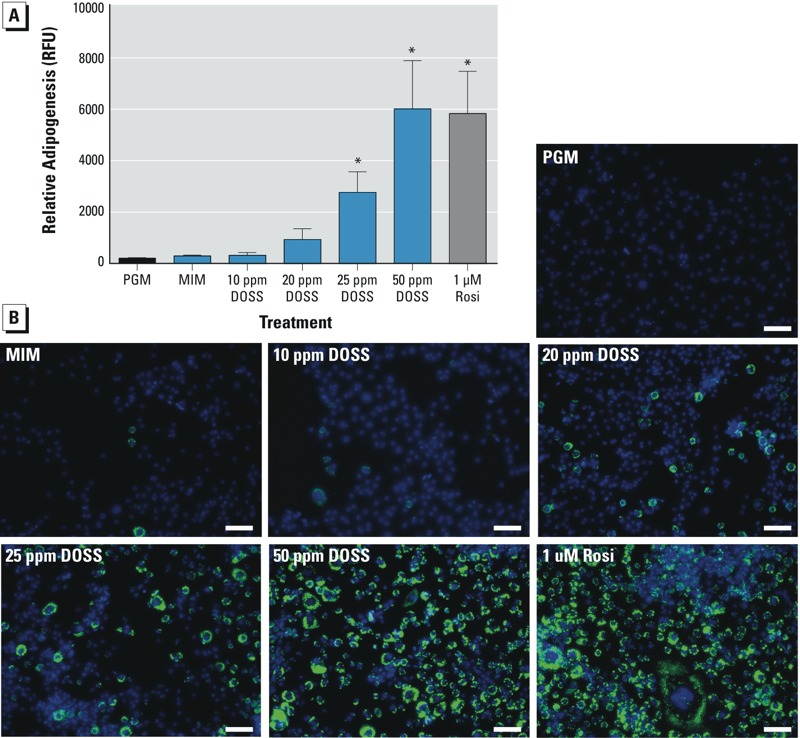
Adipogenesis following DOSS exposure *in vitro*. 3T3-L1 cells were grown to confluence over 72 hr in PGM (preadipocyte growth media). RFU, relative fluorescence units (firefly relative to renilla). Cells were either maintained in PGM or treated with MIM (minimal induction media) alone or supplemented with 10, 20, 25, or 50 ppm DOSS or 1 μM Rosi for an additional 3 days before analysis. (*A*) AdipoRed quantification of lipid accumulation as described in “Materials and Methods.” Data are expressed as mean ± SD; *n* = 5 per group (*p < 0.05 vs. MIM control). (*B*) Representative images of lipid accumulation of cells treated as in *A* (scale bar = 50 μm).

*Adipocyte differentiation* in vitro. To functionally test the obesogenic potential of DOSS, murine 3T3-L1 preadipocytes were exposed to low-level adipocyte differentiation inducers with and without DOSS, and adipogenesis was quantified by determining triacylglycerol content (Figure 7A,B). Cells were treated with MIM with and without DOSS or 1 μM Rosi (positive control). Triacylglycerol accumulation was quantified using AdipoRed staining (green) normalized to cell number (Hoechst nuclear counterstaining). Dose-dependent increases in adipogenesis were observed with DOSS treatment, with significant differences observed at 25 and 50 ppm DOSS (Figure 7A,B). In addition, mRNA expression of the preadipocyte marker Pref-1/Dlk1 decreased, whereas mRNA expression of the adipocyte marker fatty acid binding protein (Fabp4) increased dose dependently after 72 hr DOSS treatment (see Supplemental Material, Figure S6). These results demonstrate that DOSS promotes adipocyte differentiation *in vitro*.

## Discussion

Increases in general health and longevity over the past century are tributes to our knowledge of the scientific basis of good health. These impressive and hard-won gains are now threatened by an obesity epidemic (Stewart et al. 2009). Poor nutrition and lifestyle are well-established drivers of obesity. Additional contributors might include “obesogens,” compounds that alter metabolism and fat cell production, as indicated by cell culture and animal model studies (Grün and Blumberg 2006; Janesick and Blumberg 2011). Maternal–fetal obesogen exposure studies in animal models suggest that obesogens might exacerbate multiple lifelong health issues (Daftary and Taylor 2006; Janesick and Blumberg 2011; Suzuki et al. 2007) and might exacerbate obesity in future generations (Li et al. 2012; Tracey et al. 2013). Still, sound evidence of direct effects in humans remains to be determined; it is unclear whether environmental obesogens, singly or in combination, act through known metabolic and/or adipogenic mechanisms (e.g., via PPARγ) to cause clinically significant outcomes in humans, such as obesity, type 2 diabetes, insulin resistance syndrome, and polycystic ovary syndrome. Obesogens that are most prevalent in the environment, including those in widely used dispersants, consumables, and personal care products would be a good focus of such attention.

Here we have identified a novel likely obesogen, DOSS, as evidenced by PPARγ transactivation activity in a transfected cell line, activation of PPRE in transgenic reporter mice, and induction of adipogenesis in preadipocytes *in vitro*. DOSS affinity to human PPARγ receptor was also shown to be comparable with the pharmaceutical pioglitazone and an endogenous ligand arachidonic acid. In addition to being a principal component of COREXIT oil dispersant used during DWH remediation, DOSS is also used in many consumer goods, including laxatives, household cleaning products, deodorants, hair coloring, and nail polishes (DHHS 2014; EWG 2015a). That DOSS is “generally recognized as safe” and is a common additive in flavored drinks means that its use may continue to be widespread, creating the likelihood of long-term exposures [EWG 2015b; U.S. Food and Drug Administration (FDA) 1998]. Significantly, DOSS itself (Colace) is prescribed as a laxative for pregnant women, in which there is a 38% prevalence of constipation, the treatment with which could possibly affect fetal development (Jewell and Young 2001). Also, fetuses and neonates can be at greater risk to food-borne contaminants, so groups studying seafood contaminant levels suggest that the FDA revise the levels of concern for these compounds for pregnant women and children (Rotkin-Ellman et al. 2012). Although environmentally relevant DOSS exposures are not yet known, oral laxative use in pregnant women of up to 500 mg/day (88.5 kg in an average term pregnancy) would be expected to be within an order of magnitude of the dose of DOSS used in our initial mouse studies, which resulted in significant PPRE activity *in vivo* (Figure 5). Clearly, *in vivo* oral or topical dosing studies in animals at doses that are environmentally relevant are warranted to fully substantiate and understand the direct and long-term impacts of such subtoxic level exposures of this likely obesogen (Buonsante et al. 2014).

## Conclusion

The results of this study indicate that the major COREXIT dispersant component DOSS is a potential obesogen. This work indicates that DOSS might be a compound that negatively affects health and that further investigation is warranted. The subsequent identification of metabolites and biomarkers of DOSS exposure and biological consequences of exposure will aid in assessing its contribution to obesity and related health concerns.

## Supplemental Material

(1.3 MB) PDF

## References

[r1] Adedara IA, Ebokaiwe AP, Mathur PP, Farombi EO (2014). Nigerian bonny light crude oil induces endocrine disruption in male rats.. Drug Chem Toxicol.

[r2] Almeda R Wambaugh Z Wang Z Hyatt C Liu Z Buskey EJ 2013 Interactions between zooplankton and crude oil: toxic effects and bioaccumulation of polycyclic aromatic hydrocarbons. PloS One 8 e67212; doi:10.1371/journal.pone.0067212 23840628 PMC3696092

[r3] Bility MT, Thompson JT, McKee RH, David RM, Butala JH, Vanden Heuvel JP (2004). Activation of mouse and human peroxisome proliferator-activated receptors (PPARs) by phthalate monoesters.. Toxicol Sci.

[r4] Buonsante VA, Muilerman H, Santos T, Robinson C, Tweedale AC (2014). Risk assessment’s insensitive toxicity testing may cause it to fail.. Environ Res.

[r5] Calle EE (2007). Obesity and cancer.. BMJ.

[r6] Chen R, Yu X, Li L (2002). Characterization of poly(ethylene glycol) esters using low energy collision-induced dissociation in electrospray ionization mass spectrometry.. J Am Soc Mass Spectrom.

[r7] Daftary GS, Taylor HS (2006). Endocrine regulation of HOX genes.. Endocr Rev.

[r8] DHHS (U.S. Department of Health and Human Services). (2014). Household Products Database. http://householdproducts.nlm.nih.gov/cgi-bin/household/brands?tbl=chem&id=200.

[r9] El-Jamal N, Dubuquoy L, Auwerx J, Bertin B, Desreumaux P (2013). *In vivo* imaging reveals selective PPAR activity in the skin of peroxisome proliferator-activated receptor responsive element-luciferase reporter mice.. Exp Dermatol.

[r10] EWG (Environmental Working Group). (2015a). EWG’s Skin Deep® Cosmetics Database. Dioctyl Sodium Sulphosuccinate. http://www.ewg.org/skindeep/ingredient/702082/DIOCTYL_SODIUM_SULFOSUCCINATE/.

[r11] EWG. (2015b). Showing 1 to 2 of 2 Products containing Dioctyl Sodium Sulphosuccinate. http://www.ewg.org/foodscores/ingredients/9152-DioctylSodiumSulphosuccinate/search.

[r12] FDA (U.S. Food and Drug Administration). (1998). Cytec Industries, Inc.—GRAS Notification for Dioctyl Sodium Sulfosuccinate; Our File No. CY04825/05 to Laura M. Tarantino, Acting Director, FDA, Washington, DC, from Law Offices, Keller and Heckman, LLP, Washington, DC. http://www.fda.gov/ucm/groups/fdagov-public/@fdagov-foods-gen/documents/document/ucm264416.pdf.

[r13] Forman BM, Tontonoz P, Chen J, Brun RP, Spiegelman BM, Evans RM (1995). 15-Deoxy-Δ^12^,^14^-prostaglandin *J*_2_ is a ligand for the adipocyte determination factor PPARγ.. Cell.

[r14] Grün F, Blumberg B (2006). Environmental obesogens: organotins and endocrine disruption via nuclear receptor signaling.. Endocrinology.

[r15] Hemmer MJ, Barron MG, Greene RM (2011). Comparative toxicity of eight oil dispersants, Louisiana sweet crude oil (LSC), and chemically dispersed LSC to two aquatic test species.. Environ Toxicol Chem.

[r16] Hvattum E, Yip WL, Grace D, Dyrstad K (2012). Characterization of polysorbate 80 with liquid chromatography mass spectrometry and nuclear magnetic resonance spectroscopy: specific determination of oxidation products of thermally oxidized polysorbate 80.. J Pharm Biomed Anal.

[r17] Janesick A, Blumberg B (2011). Minireview: PPARγ as the target of obesogens.. J Steroid Biochem Mol Biol.

[r18] Jewell DJ Young G 2001 Interventions for treating constipation in pregnancy. Cochrane Database Syst Rev 2 CD001142; doi:10.1002/14651858.CD001142 11405974

[r19] Judson RS, Martin MT, Reif DM, Houck KA, Knudsen TB, Rotroff DM (2010). Analysis of eight oil spill dispersants using rapid, *in vitro* tests for endocrine and other biological activity.. Environ Sci Technol.

[r20] Kirchner S, Kieu T, Chow C, Casey S, Blumberg B (2010). Prenatal exposure to the environmental obesogen tributyltin predisposes multipotent stem cells to become adipocytes.. Mol Endocrinol.

[r21] Kujawinski EB, Kido Soule MC, Valentine DL, Boysen AK, Longnecker K, Redmond MC (2011). Fate of dispersants associated with the Deepwater Horizon oil spill.. Environ Sci Technol.

[r22] Li X Pham HT Janesick AS Blumberg B 2012 Triflumizole is an obesogen in mice that acts through peroxisome proliferator activated receptor gamma (PPARγ). Environ Health Perspect 120 1720 1726; doi:10.1289/ehp.1205383 23086663 PMC3548286

[r23] Mathew J, Schroeder DL, Zintek LB, Schupp CR, Kosempa MG, Zachary AM (2012). Dioctyl sulfosuccinate analysis in near-shore Gulf of Mexico water by direct-injection liquid chromatography–tandem mass spectrometry.. J Chromatogr A.

[r24] National Research Council. (2011). Guide for the Care and Use of Laboratory Animals, 8th ed.

[r25] Newbold RR, Padilla-Banks E, Snyder RJ, Jefferson WN (2005). Developmental exposure to estrogenic compounds and obesity.. Birth Defects Res A Clin Mol Teratol.

[r26] Ogden CL, Carroll MD, Curtin LR, McDowell MA, Tabak CJ, Flegal KM (2006). Prevalence of overweight and obesity in the United States, 1999–2004.. JAMA.

[r27] Ramachandran SD, Hodson PV, Khan CW, Lee K (2004). Oil dispersant increases PAH uptake by fish exposed to crude oil.. Ecotoxicol Environ Saf.

[r28] Ramirez CE, Batchu SR, Gardinali PR (2013). High sensitivity liquid chromatography tandem mass spectrometric methods for the analysis of dioctyl sulfosuccinate in different stages of an oil spill response monitoring effort.. Anal Bioanal Chem.

[r29] Rico-Martínez R, Snell TW, Shearer TL (2013). Synergistic toxicity of Macondo crude oil and dispersant Corexit 9500A® to the *Brachionus plicatilis* species complex (Rotifera).. Environ Pollut.

[r30] Rotkin-Ellman M Wong KK Solomon GM 2012 Seafood contamination after the BP Gulf oil spill and risks to vulnerable populations: a critique of the FDA risk assessment. Environ Health Perspect 120 157 161; doi:10.1289/ehp.1103695 21990339 PMC3279436

[r31] Rundle A, Hoepner L, Hassoun A, Oberfield S, Freyer G, Holmes D (2012). Association of childhood obesity with maternal exposure to ambient air polycyclic aromatic hydrocarbons during pregnancy.. Am J Epidemiol.

[r32] Singh U, Marks BD, Eliason HC, Stafslien DK, Wilkinson JM, De Rosier T, et al. (2008). New fluorescence-based assays for identification and characterization of selective PPARα, δ(β), and γ ligands [Abstract].. In: Proceedings of the 235th ACS National Meeting. 6–10 April 2008.

[r33] Somm E Schwitzgebel VM Toulotte A Cederroth CR Combescure C Nef S 2009 Perinatal exposure to bisphenol A alters early adipogenesis in the rat. Environ Health Perspect 117 1549 1555; doi:10.1289/ehp.11342 20019905 PMC2790509

[r34] Stahlhut RW van Wijngaarden E Dye TD Cook S Swan SH 2007 Concentrations of urinary phthalate metabolites are associated with increased waist circumference and insulin resistance in adult U.S. males. Environ Health Perspect 115 876 882; doi:10.1289/ehp.9882 17589594 PMC1892109

[r35] Stewart ST, Cutler DM, Rosen AB (2009). Forecasting the effects of obesity and smoking on U.S. life expectancy.. N Engl J Med.

[r36] Suzuki A, Urushitani H, Sato T, Kobayashi T, Watanabe H, Ohta Y (2007). Gene expression change in the Müllerian duct of the mouse fetus exposed to diethylstilbestrol *in utero*.. Exp Biol Med (Maywood).

[r37] Toth PM, Naruhn S, Pape VF, Dörr SM, Klebe G, Müller R (2012). Development of improved PPARβ/δ inhibitors.. ChemMedChem.

[r38] Tracey R, Manikkam M, Guerrero-Bosagna C, Skinner MK (2013). Hydrocarbons (jet fuel JP-8) induce epigenetic transgenerational inheritance of obesity, reproductive disease and sperm epimutations.. Reprod Toxicol.

[r39] Zhang R, Wang Y, Tan L, Zhang HY, Yang M (2012). Analysis of polysorbate 80 and its related compounds by RP-HPLC with ELSD and MS detection.. J Chromatogr Sci.

