# Supplementary material for: Effects of Crude Oil/Dispersant Mixture and Dispersant Components on PPARγ Activity in Vitro and in Vivo: Identification of Dioctyl Sodium Sulfosuccinate (DOSS; CAS #577-11-7) as a Probable Obesogen
Source: Environ Health Perspect. 2015 Jul 2;124(1):112–9. doi: 10.1289/ehp.1409672 (PMC4710608; doi:10.1289/ehp.1409672)
Supplement: (1.3 MB) PDF [file ehp.1409672.s001.acco.pdf]

Environ Health Perspect
DOI: 10.1289/ehp.1409672

Note to Readers: *EHP* strives to ensure that all journal content is accessible to all readers. However, some figures and Supplemental Material published in *EHP* articles may not conform to 508 standards due to the complexity of the information being presented. If you need assistance accessing journal content, please contact ehp508@niehs.nih.gov. Our staff will work with you to assess and meet your accessibility needs within 3 working days.

Supplemental Material

Effects of Crude Oil/Dispersant Mixture and Dispersant Components on PPARγ Activity *in Vitro* and *in Vivo*: Identification of Dioctyl Sodium Sulfosuccinate (DOSS; CAS #577-11-7) as a Probable Obesogen

Alexis M. Temkin, Robert R. Bowers, Margaret E. Magaletta, Steven Holshouser, Adriana Maggi, Paolo Ciana, Louis J. Guillette, John A. Bowden, John R. Kucklick, John E. Baatz, and Demetri D. Spyropoulos

Table of Contents

Table S1. Primers used for qPCR analysis of 3T3-L1 cells.

Figure S1. PPARγ ligand binding activity of DWAF in a GAL4-UAS system. Dilutions of mixtures were prepared, HEK293T/17 cells were transfected, exposed in triplicate for 18 hours, and luciferase activities were measured as detailed in *Materials and Methods*. Dose dependent ligand binding activities were not detected in any of the DWAF dilutions tested. Data are expressed as Mean ± SD; n = 3 per group (no statistically significant differences).

Figure S2. Detection of prevalent COREXIT components Tween 80 and DOSS in the 50:50 ethanol:water fraction. (Top) Total ion chromatogram in full scan positive mode of the CWAF ethanol/water SPE extractable fraction. Inset A, corresponding to peak at 5.33 min, displays a positive mode precursor ion scan of *m/z* 309.3 that produces a mass spectral pattern (sorbitan monooleates with 16 – 27 polyoxy-ethylene units) previously found in Tween 80 (Zhang et al. 2012). (Bottom) Total ion chromatogram in full scan negative mode of the CWAF ethanol/water SPE extractable fraction. Inset B, corresponding to peak at 2.45 min, displays a product ion scan

for *m/z* 421.0. Examining the fragmentation profile, noting the fragment ion *m/z* 81.0, indicates the presence of DOSS, as previously reported (Mathew et al. 2012; Ramirez et al. 2013).

Figure S3. Molecular modeling of PPARγ with COREXIT components Span 80, Tween 80 and dioctyl sodium sulfosuccinate (DOSS). A) Average E Score for COREXIT components analyzed for PPARγ ligand binding using MOE as in *Materials and Methods*. B) DOSS modeled in the PPARγ ligand-binding pocket.

Figure S4. PPARγ activity of COREXIT components other than DOSS. Dilutions of mixtures were prepared, HEK293T/17 cells were transfected, exposed in triplicate for 18 hours, and luciferase activities were measured as detailed in *Materials and Methods*. Dose-dependent ligand binding activities were not detected in any of the dilutions tested for A) Span 80 or C) ICP:Propylene Glycol (PG). Modest ligand binding activity was detected only at the highest dose of B) Tween 80 (50ppm). Robust dose-dependent ligand binding activities were detected in D) ICP:PG:DOSS (4 ppm and 8 ppm). Collectively, these results indicate that DOSS is the principle obesogen in COREXIT. Data are expressed as Mean ± SD; n = 3 per group (* $p < 0.05$ versus untreated controls).

Figure S5. DOSS has Human PPARγ, PPARα, PPARβ/δ, and RXRα transactivation activity. Dilutions of mixtures and positive controls were prepared, HEK293T/17 cells were transfected, exposed in triplicate for 18 h, and luciferase activities were measured. A) PPARγ, B) PPARα, C) PPARβ/δ and D) RXRα. Dose-dependent transactivation activities by DOSS were detected for PPARγ and PPARβ/δ. Data are expressed as Mean ± SD; n = 3 per group (* $p < 0.05$ versus not treatment control).

Figure S6. Dlk1 and Fabp4 mRNA expression in 3T3-L1 cells at day three of adipogenesis. 3T3-L1 cells were treated for 72 h as described in *Materials and Methods* and qPCR was performed as described in *Supplemental Materials and Methods*. Data are represented as a fold change in expression over the housekeeping gene HPRT for A) preadipocyte marker Pref-1/Dlk1 and B) adipocyte marker Fabp4 (* $p < 0.05$ versus MIM control, n = 6 per group).

Table S1. Primers used for qPCR analysis of 3T3-L1 cells.

Species	Gene	Forward Primer	Reverse Primer
Mouse	Hprt	AGGCCAGACTTTGTTGGATTTG	TTCAACTTGCGCTCATCTTAGG
Mouse	Pref-1/Dlk1	CCTGGCTGTGTCAATGGAGT	CAAGTTCCATTGTTGGCGCA
Mouse	Fabp4	ATGTGTGATGCCTTTGTGGGA	GATGATCATGTTGGGCTTGGC

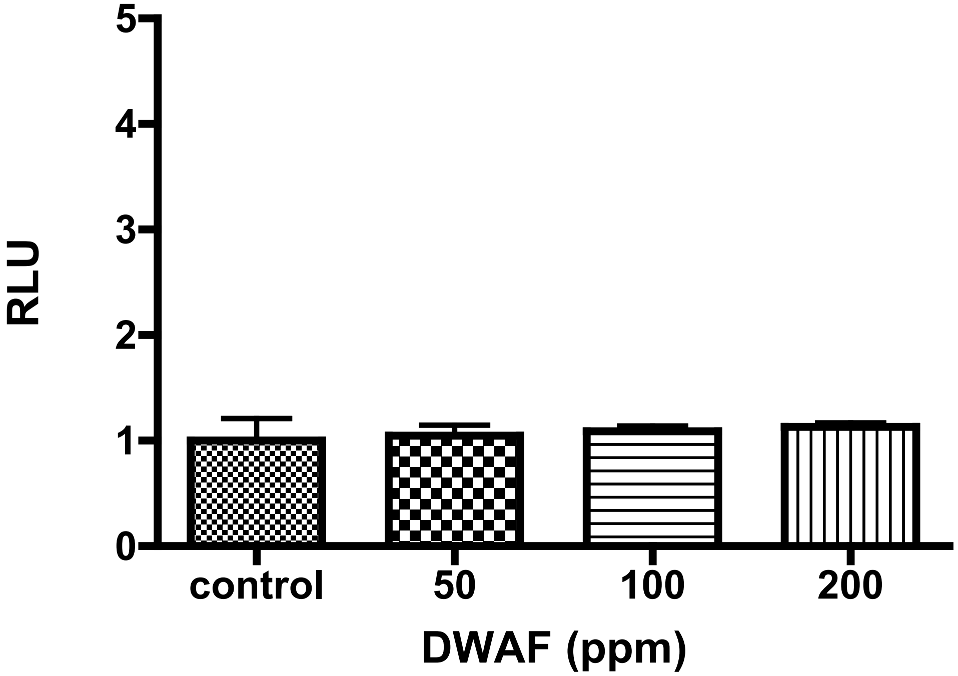

Figure S1. PPARγ ligand binding activity of DWAF in a GAL4-UAS system. Dilutions of mixtures were prepared, HEK293T/17 cells were transfected, exposed in triplicate for 18 hours, and luciferase activities were measured as detailed in *Materials and Methods*. Dose dependent ligand binding activities were not detected in any of the DWAF dilutions tested. Data are expressed as Mean ± SD; n = 3 per group (no statistically significant differences).

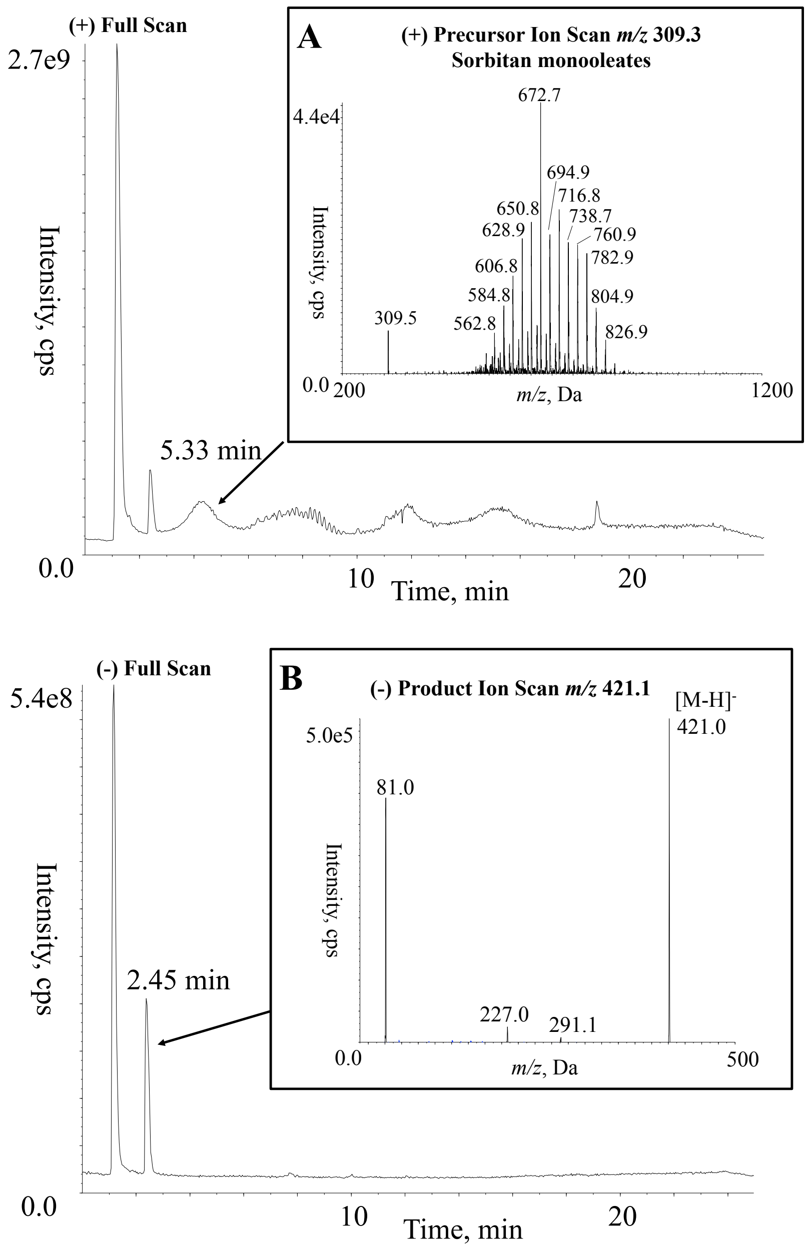

Figure S2. Detection of prevalent COREXIT components Tween 80 and DOSS in the 50:50 ethanol:water fraction. (Top) Total ion chromatogram in full scan positive mode of the CWAF ethanol/water SPE extractable fraction. Inset A, corresponding to peak at 5.33 min, displays a positive mode precursor ion scan of *m/z* 309.3 that produces a mass spectral pattern (sorbitan monooleates with 16 – 27 polyoxy-ethylene units) previously found in Tween 80 (Zhang et al. 2012). (Bottom) Total ion chromatogram in full scan negative mode of the CWAF ethanol/water SPE extractable fraction. Inset B, corresponding to peak at 2.45 min, displays a product ion scan for *m/z* 421.0. Examining the fragmentation profile, noting the fragment ion *m/z* 81.0, indicates the presence of DOSS, as previously reported (Mathew et al. 2012; Ramirez et al. 2013).

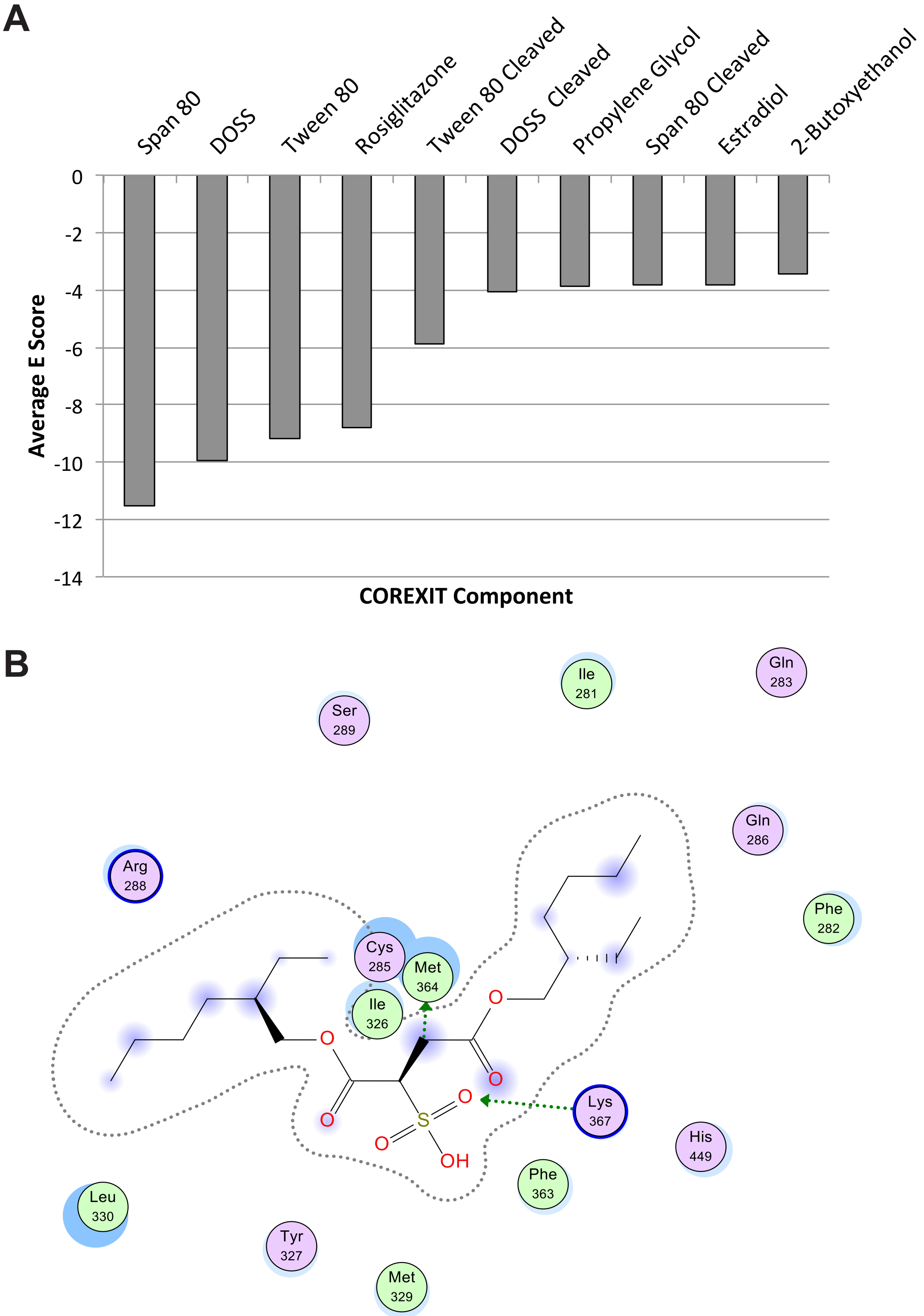

Figure S3. Molecular modeling of PPARγ with COREXIT components Span 80, Tween 80 and dioctyl sodium sulfosuccinate (DOSS). A) Average E Score for COREXIT components analyzed for PPARγ ligand binding using MOE as in *Materials and Methods*. B) DOSS modeled in the PPARγ ligand-binding pocket.

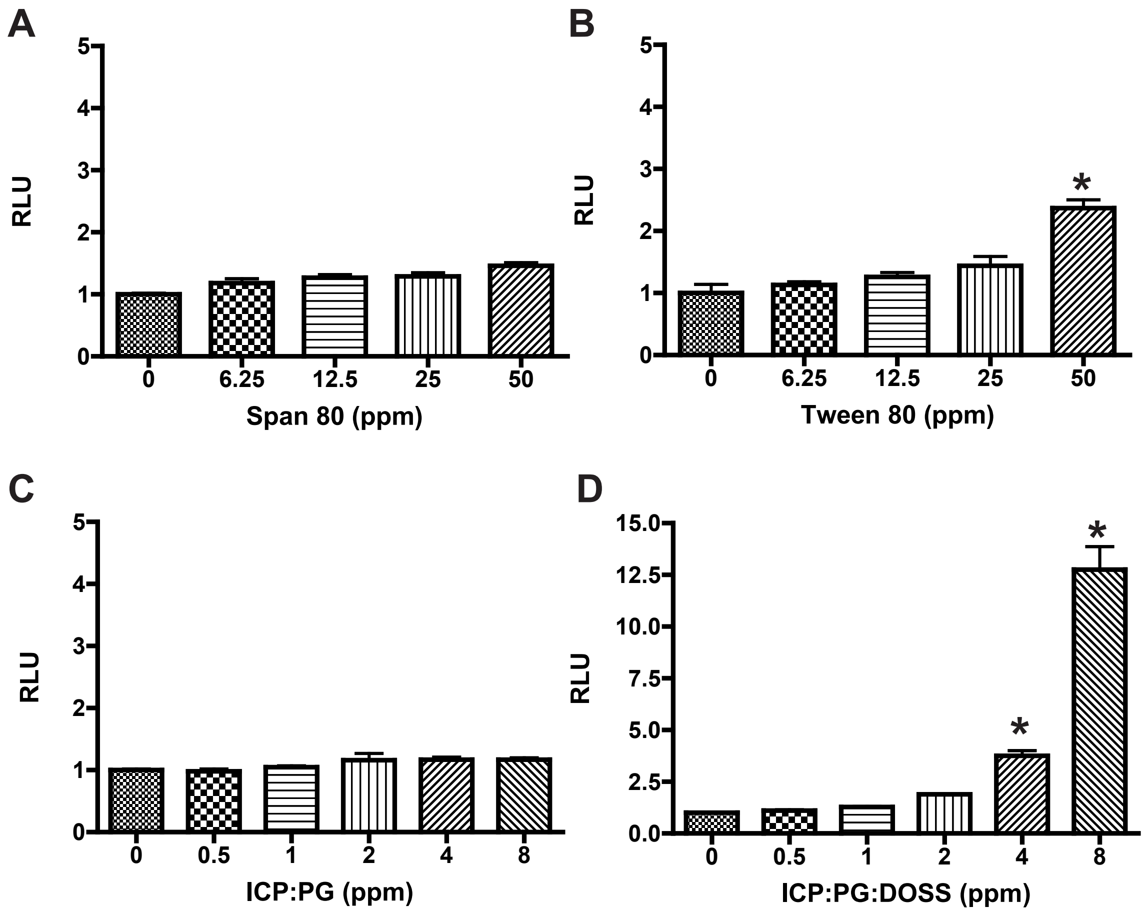

Figure S4. PPARγ activity of COREXIT components other than DOSS. Dilutions of mixtures were prepared, HEK293T/17 cells were transfected, exposed in triplicate for 18 hours, and luciferase activities were measured as detailed in *Materials and Methods*. Dose-dependent ligand binding activities were not detected in any of the dilutions tested for A) Span 80 or C) ICP:Propylene Glycol (PG). Modest ligand binding activity was detected only at the highest dose of B) Tween 80 (50ppm). Robust dose-dependent ligand binding activities were detected in D) ICP:PG:DOSS (4 ppm and 8 ppm). Collectively, these results indicate that DOSS is the principle obesogen in COREXIT. Data are expressed as Mean ± SD; n = 3 per group (* $p < 0.05$ versus untreated controls).

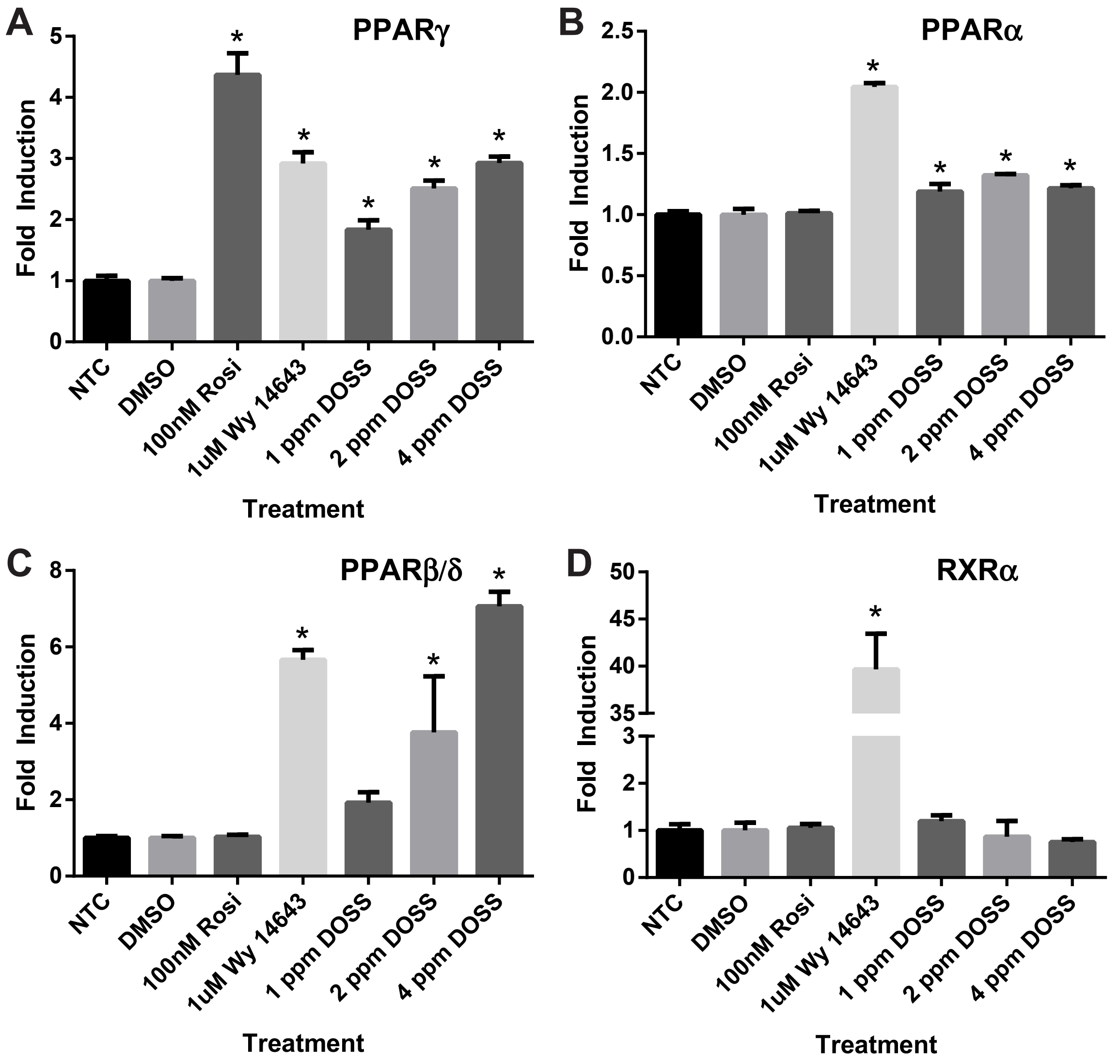

Figure S5. DOSS has Human PPARγ, PPARα, PPARβ/δ, and RXRα transactivation activity. Dilutions of mixtures and positive controls were prepared, HEK293T/17 cells were transfected, exposed in triplicate for 18 h, and luciferase activities were measured. A) PPARγ, B) PPARα, C) PPARβ/δ and D) RXRα. Dose-dependent transactivation activities by DOSS were detected for PPARγ and PPARβ/δ. Data are expressed as Mean ± SD; n = 3 per group (* $p < 0.05$ versus not treatment control).

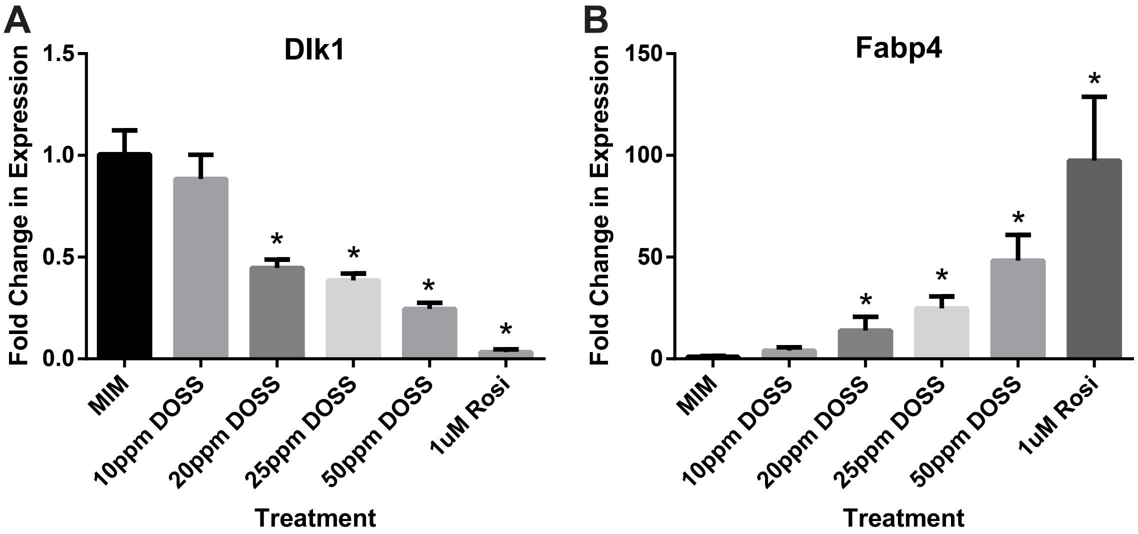

Figure S6. Dlk1 and Fabp4 mRNA expression in 3T3-L1 cells at day three of adipogenesis. 3T3-L1 cells were treated for 72 h as described in *Materials and Methods* and qPCR was performed as described in *Supplemental Materials and Methods*. Data are represented as a fold change in expression over the housekeeping gene HPRT for A) preadipocyte marker Pref-1/Dlk1 and B) adipocyte marker Fabp4 (* $p < 0.05$ versus MIM control, n = 6 per group).
